# Molecular characterization and pathogenicity stratification of *YWHAG* missense variants in developmental and epileptic encephalopathy

**DOI:** 10.3389/fgene.2026.1875740

**Published:** 2026-10-05

**Authors:** Wen-Hui Liu, Juan Zhou, Zhong-Yan Zhu, Qian-Ru Wen, Si-Qi Zhang, Yan Ding, Heng Meng

**Affiliations:** 1 Department of Neurology, The First Affiliated Hospital & Clinical Neuroscience Institute of Jinan University, Guangzhou, China; 2 Department of Neurology, The First People’s Hospital of Chenzhou, Chenzhou, Hunan, China; 3 The Sixth Affiliated Hospital of Jinan University, Dongguan, Guangdong, China

**Keywords:** developmental and epileptic encephalopathy, missense variant, pathogenicity assessment, protein‒protein interaction, spatial-temporal expression, YWHAG

## Abstract

**Background:**

Variants in *YWHAG* cause severe developmental and epileptic encephalopathy (DEE) with early-onset seizures. Rapid and precise clinical classification of its missense variants presents a substantial challenge. This study sought to explore YWHAG spatiotemporal expression-pathogenicity correlation and reliable pathogenicity indicators for its variants to promote clinical genetic diagnosis.

**Methods:**

In this study, 55 *YWHAG* missense variants were curated, including 17 pathogenic/likely pathogenic variants that met ACMG guideline criteria and 38 benign variants from gnomAD. We performed protein structure modeling, spatiotemporal expression profiling, single-cell transcriptome analysis, PPI network construction, and functional enrichment analysis. We also evaluated 34 pathogenicity prediction tools using accuracy, balanced accuracy, AUC, and MCC.

**Results:**

Protein structure modeling revealed that hydrogen bond alterations occurred in both pathogenic and benign *YWHAG* variants, which could not reliably distinguish the two groups. YWHAG was highly expressed in the central nervous system with three distinct developmental peaks, shifting from both excitatory and inhibitory neurons to predominantly excitatory neurons during cerebral organoid maturation. The YWHAG protein regulated the MAPK, PI3K-Akt, Hippo pathways and cell cycle progression. Among the 34 evaluated tools, deep learning and ensemble algorithms outperformed traditional conservation-based predictors, with MetaSVM showing the best overall performance, MetaLR achieving excellent specificity and ESM1b attaining the highest AUC value.

**Conclusion:**

Meta-predictors may effectively predict *YWHAG* missense variant pathogenicity, and *YWHAG* spatiotemporal expression aligns with its DEE pathogenic role. These computational tools may provide reliable support for the pathogenicity assessment of *YWHAG* variants in clinical genetic diagnosis.

## 1 Introduction

Developmental and epileptic encephalopathy (DEE) is a severe neurodevelopmental disorder characterized by early-onset, refractory seizures and profound developmental impairment, resulting in substantial lifelong disability and healthcare burden (Poke et al., 2023; Guerrini et al., 2023). While etiologies are diverse, encompassing structural, metabolic, and infectious causes, genetic factors are increasingly recognized as the predominant driver, with over 112 genes now definitively linked to DEEs, including *YWHAG* (Tabarki, 2024).

*YWHAG* is located on chromosome 7q11.23 and encodes tyrosine 3-monooxygenase/tryptophan 5-monooxygenase activation protein gamma, a member of the highly conserved 14-3-3 family. As a scaffold protein, it is involved in the regulation of multiple cellular signaling pathways, including critical processes such as neuronal migration, synaptic plasticity, ion channel regulation, and protein–protein interactions, and is particularly highly expressed in the central nervous system (Guella et al., 2017; Skoulakis and Davis, 1998). In recent years, numerous studies have confirmed that heterozygous pathogenic variants in *YWHAG* are the monogenic cause of developmental and epileptic encephalopathy 56 (DEE56, OMIM #617665), which is mainly characterized by early-onset refractory epilepsy in infancy, global developmental delay, intellectual disability, and autistic spectrum features, with a phenotypic spectrum ranging from mild myoclonic epilepsy with febrile seizures to severe DEE (Wachi et al., 2016; Cornell and Toyo-Oka, 2017; Ye et al., 2021).

In this study, we systematically collected and analyzed 55 *YWHAG* missense variants, comprising 17 pathogenic or likely pathogenic variants rigorously classified according to ACMG criteria and 38 benign control variants sourced from gnomAD, with benign/likely benign classifications annotated in ClinVar. By integrating protein structural modeling, spatiotemporal and single-cell expression profiling, protein–protein interaction network analysis, and a comprehensive benchmarking of 34 *in silico* pathogenicity prediction tools, we explored the molecular mechanisms underlying *YWHAG* missense variants in the pathogenesis of developmental and epileptic encephalopathy. Performance evaluation revealed that modern deep learning-based and structure-integrated algorithms (such as ESM1b, MetaSVM, AlphaMissense, and MetaLR) substantially outperformed traditional sequence conservation-based methods, achieving markedly higher specificity, overall accuracy, and Matthew’s correlation coefficient while preserving excellent sensitivity. These superior tools better captured subtle structural perturbations affecting the ligand-binding groove and dimer interface of the protein. Our findings provide gene-specific computational evidence to support more accurate clinical interpretation of *YWHAG* missense variants and contribute to improving diagnostic strategies for genetic developmental and epileptic encephalopathies.

## 2 Materials and methods

### 2.1 Variants collection

To assess the performance of *in silico* tools for predicting the pathogenicity of *YWHAG* missense variants, we categorized variants into two groups: disease-associated variants and control variants. Disease-related variants were retrieved from the Human Gene Mutation Database (HGMD) and PubMed using the search term *YWHAG* AND (variant OR mutation), and were strictly screened against four criteria: (1) supported by sufficient clinical evidence for disease diagnosis; (2) other known disease-causing genes were largely excluded; (3) the origin of the variant was clearly defined; (4) pathogenicity classification was consistent with the 2015 ACMG/AMP standards and guidelines for sequence variant interpretation. Pathogenicity classification was independently performed by two board-certified clinical geneticists, and discrepancies were resolved through consensus review. The full ACMG/AMP evidence codes for each disease-associated variant, together with brief supporting rationales, are summarized in Supplementary Table S1 in the supplementary materials.

Control variants were retrieved from gnomAD, with benign/likely benign classifications cross-annotated from interoperable ClinVar entries. As ClinVar lacks complete population frequency data, gnomAD v2.1 was used as the primary source, and only variants with minor allele frequency <0.01 were retained. The non-neurological control variants were selected according to the following criteria: missense variants from the non-neuro subset of gnomAD v2.1 that are classified as Benign/Likely benign. All data were verified as of 1 April 2026.

### 2.2 In silico prediction

Thirty-four bioinformatics tools were selected for the present study, based on their common clinical usage and validated efficacy in predicting the functional consequences of missense variants. Tool selection adhered to three core criteria: first, each tool was either cited in the ACMG variant interpretation guidelines, recommended by clinical genetics consortia, or provided in major variant annotation platforms including Franklin and VarSome; second, clear and officially recommended score thresholds for pathogenicity classification were available; third, valid scores could be generated for at least 80% of the variants included in this work. To ensure result reproducibility, all pathogenicity prediction scores for the 34 evaluated tools were retrieved from the dbNSFP database (dbnsfp.org), specifically via the dbNSFP v5.4c Public Variant Browser (https://query.dbnsfp.org/). (Ng and Henikoff, 2001; Vaser et al., 2016; Adzhubei et al., 2010; Schwarz et al., 2014; Reva et al., 2011; Choi et al., 2012; Dong et al., 2015; Li et al., 2022; Jagadeesh et al., 2016; Pejave et al., 2020; Sundaram et al., 2018; Raimondi et al., 2017; Feng, 2017; Alirezaie et al., 2018; Ma et al., 2019; Cheng et al., 2023; Rogers et al., 2018), And and the exact model version and original training-set description of each predictor as included in dbNSFP v5.4c are summarized in Supplementary Table S3.

In this study, a two-step threshold strategy was adopted to achieve two independent and complementary research objectives while eliminating potential analytical bias. First, the default thresholds recommended in the literature were used to conduct direct benchmark comparisons of the 34 algorithms, and a unified general standard ensures the fairness, consistency, and reproducibility of performance evaluations across algorithms, since different tools have inherent differences in their scoring systems and cannot be directly compared using their respective gene-specific thresholds. Second, gene-specific optimized cut-offs were further derived to serve as an exploratory reference for clinical interpretation, as universal default thresholds are designed for pan-gene application and may exhibit suboptimal performance when applied to rare disease-associated genes. These optimized cut-offs were calculated using Youden’s Index for each high-performance algorithm identified in the benchmark analysis, aimed at balancing sensitivity and specificity for *YWHAG* variant discrimination within our cohort. Of note, these cut-offs represent data-driven, gene-specific values tailored to the current dataset, and have not yet undergone independent calibration or validation in external cohorts. As such, they may be regarded as preliminary reference values to inform PP3/BP4 evidence weighting, and are not intended to serve as formally validated thresholds for definitive ACMG/AMP evidence strength assignment.

### 2.3 Evaluation of predictive performance

To assess the predictive performance of the *in silico* tools, we adopted the same evaluation framework as described in our previous study (Liu et al., 2026; Gu et al., 2025; Wang et al., 2026). We determined true positives (TP), true negatives (TN), false positives (FP), and false negatives (FN). We then computed a series of metrics including accuracy, sensitivity, specificity, positive predictive value (PPV), negative predictive value (NPV), F-score, and Matthews correlation coefficient (MCC). The F score was defined as $\frac{2\mathrm{PR}}{P+R}$ , where precision (P) = $\frac{TP}{TP+FP}$ and recall (R) = $\frac{TP}{TP+FN}$ . The Matthews correlation coefficient (MCC) score was calculated using the following equation $\frac{TP\times TN-FP\times FN}{\sqrt{\left(TP+FP\right)\left(TP+FN\right)\left(TN+FP\right)\left(TN+FN\right)}}$ , with the score ranging from −1 to 1; −1 indicates a completely wrong binary classifier, while 1 indicates a completely correct binary classifier.

ROC curves were generated for each prediction tool. Disease-associated variants were defined as gold-standard positive cases, while control missense variants without related phenotypes were treated as gold-standard negative cases. The area under the curve (AUC) was computed and reported for each tool.

To assess the robustness of performance estimates and mitigate over-optimistic bias arising from threshold derivation on the same dataset, bootstrap resampling with 1000 iterations was performed. In each iteration, 55 variants were randomly sampled with replacement from the original dataset. Gene-specific optimal thresholds were recalculated using the Youden’s index method on each bootstrap sample, and all performance metrics were recomputed accordingly. The bootstrap mean, standard deviation, and 95% confidence interval for each metric were derived from the valid iterations. All statistical analyses were performed at a two-sided significance level of 0.05.

### 2.4 Damaging effects of variants

The wild-type structure of human *YWHAG* was directly retrieved from the AlphaFold Protein Structure Database (https://alphafold.ebi.ac.uk/entry/P61981) as the baseline for structural comparison. The specific model is AF-P61981-F1 (Version v6, Google DeepMind dataset), corresponding to the canonical UniProt sequence P61981 (247 amino acids). This model has an average pLDDT score of 94.19, with 89.1% of residues classified as “Very high” confidence, ensuring high reliability of the core domain structure. Single amino acid substitutions were introduced into the wild-type sequence, with local structural adjustments made based on the mutated residue, while the overall protein backbone conformation was maintained. Hydrogen bond changes were defined as alterations in bond number.

### 2.5 Spatial-temporal expression of *YWHAG*

The spatial expression pattern of *YWHAG* in various adult non-diseased tissues was analyzed using data from the Genotype-Tissue Expression (GTEx) portal (https://www.gtexportal.org/home/). The observed spatiotemporal expression pattern was consistent with our previous study (Luo et al., 2025a; Luo et al., 2025b). Expression profiles of *YWHAG* across diverse organs and developmental stages were analyzed using datasets from the Evo-devo mammalian organ online resource (https://apps.kaessmannlab.org/evodevoapp/) (Cardoso-Moreira et al., 2019). To investigate the spatiotemporal expression pattern of *YWHAG*, the BrainSpan database (http://www.brainspan.org/) was used in this study. This database covers human brain development spanning from 8 post-conceptional weeks to 40 years of age.

### 2.6 Single-cell expression of *YWHAG*

YWHAG expression across distinct developmental stages was analyzed using data from developing cerebral organoids and the normal adult human brain. Single-cell expression profiles of *YWHAG* in undirected cerebral organoids at one and 5 months were obtained from the Human Organoid Single-Cell Browser (Wang et al., 2022).

### 2.7 Protein‒protein interaction (PPI) and coexpression correlation analyses

The protein-protein interaction (PPI) network of *YWHAG* was constructed using the STRING database (https://string-db.org/) with *Homo sapiens* (NCBI taxonomy ID: 9606) as the reference organism. *YWHAG* was set as the seed gene to retrieve its direct first-shell functional interactors. Interactions were evaluated by the STRING combined confidence score, which integrates evidence from seven independent channels: neighborhood, gene fusion, co-occurrence, co-expression, experimental validation, curated databases, and text mining. The final network contains 21 protein nodes and 131 interaction edges, with an average node degree of 12.5 and an average local clustering coefficient of 0.845. The network shows statistically significant PPI enrichment (enrichment p-value <1.0e-16), indicating non-random biological connectivity among the included proteins.

Functional enrichment analysis was performed using the built-in STRING enrichment pipeline. The whole human genome was used as the statistical background for all enrichment calculations. All raw p-values were adjusted via the Benjamini–Hochberg procedure to control the false discovery rate (FDR), and functional terms with an adjusted FDR ≤0.05 were defined as significantly enriched. Enrichment analyses were conducted across multiple annotation categories, including tissue expression profiles (TISSUES), Gene Ontology (biological process, molecular function), KEGG pathways, and Reactome pathways.

### 2.8 Statistical analysis

Statistical analyses were performed using GraphPad Prism version 10.4.2. The distribution of structurally modified residues between pathogenic and benign *YWHAG* variants was compared using Fisher’s exact test for 2 × 2 contingency tables.

For each of the 34 *in silico* prediction tools, differences in continuous pathogenicity scores between disease-associated and benign control variants were assessed using the Mann–Whitney U test (Wilcoxon rank-sum test). Benjamini–Hochberg false discovery rate (FDR) correction was applied to adjust P values across all 34 between-group comparisons to account for multiple testing. A two-sided P-value <0.05 was considered statistically significant. Receiver operating characteristic (ROC) curves were constructed for each prediction tool, and the area under the curve (AUC) with corresponding 95% confidence interval (CI) was calculated using the DeLong non-parametric method. Pairwise comparisons of AUC values between algorithms were also performed using the DeLong test. A complete-case analysis strategy was adopted for missing predictor scores: variants with unavailable scores for a given algorithm were excluded only from the analysis of that specific tool. All included algorithms had a valid score call rate of ≥80% across the full variant set, in accordance with the tool inclusion criteria specified in Section 2.2.

## 3 Results

### 3.1 Clinical features of *YWHAG* missense variants

In this study, the dataset of missense variants in the *YWHAG* gene included 17 disease-associated variants (N = 17) and 38 control variants (N = 38). Pathogenic variants in the *YWHAG* gene are predominantly associated with DEE. These 17 pathogenic missense variants were including the following: p.Glu15Ala, p.Arg57Gly, p.Arg57Cys, p.Arg57His, p.Lys125Glu, p.Asp129Glu, p.Arg132Gly, p.Arg132Cys, p.Arg132His, p.Tyr133Asn, p.Tyr133Ser, p.Tyr133Cys, p.Leu177Ile, p.Asn178Asp, p.Ala193Val, p.Ser180Tyr, and p.Glu207Lys (Supplementary Table S2).

A total of 56 clinical cases harboring these 17 pathogenic missense variants in the YWHAG gene were meticulously curated from reliable sources: pathogenic variants were obtained from the Human Gene Mutation Database (HGMD) and PubMed, supported by robust clinical data, whereas benign variants were retrieved from the Genome Aggregation Database (gnomAD), ensuring their non-deleterious nature. The patients were mainly diagnosed with DEE. The age at seizure onset ranged from 1 month to 2 years, with a median of approximately 12 months. The core seizure types were predominantly generalized tonic-clonic seizures (76.8%), myoclonic seizures (66.1%), and atypical absences (25.0%), with focal seizures occurring in 23.2% of patients. EEG mainly showed generalized discharges, with some cases accompanied by multifocal/focal discharges or hypsarrhythmia. Brain magnetic resonance imaging (MRI) was normal in the majority of patients. Abnormal MRI findings mainly included enlarged bilateral ventricles, delayed white matter myelination, cortical atrophy, subtle white matter signal changes, venous anomaly, arachnoid cyst, frontal lobe hyperintensity, and non-specific FLAIR hyperintensities. Antiepileptic drugs (AEDs) were mainly used in combination, such as valproic acid (VPA), levetiracetam (LEV), lamotrigine (LTG), and clobazam (CLB); some patients could achieve long-term seizure freedom in the later stage, but seizure control remained unsatisfactory in most patients. Almost all patients exhibited moderate-to-severe developmental delay or intellectual disability, commonly accompanied by ataxia, tremor, and hypotonia. Approximately one-third to one-half of patients had autism spectrum disorder, attention deficit hyperactivity disorder, or aggressive behavior. In addition, 40%–50% presented with mild facial dysmorphism, including hypertelorism, epicanthus, prominent forehead, flat nasal bridge, and ear abnormalities (Supplementary Table S2).

Structural alterations of the variants were evaluated by means of protein modeling with I-TASSER and structural visualization using PyMOL. Our results showed that at least one variant in each case was predicted to disrupt or alter hydrogen bonds with surrounding residues (Figures 1, 2). Specifically, among the 17 disease-associated variants, 11 (64.7%) exhibited hydrogen bond alterations. In the 38 control variants, 20 (52.6%) showed changes in hydrogen bond interactions. These findings indicate that there is no significant difference between pathogenic variants and control variants in terms of their ability to induce hydrogen bond perturbations. Additionally, we mapped the positions of all 55 variants against the modified residue annotations from the UniProt database. As shown in Figure 3, the proportion of variants affecting modified residues did not differ statistically between the pathogenic and benign groups (*P* = 0.892). This result suggests that disruption of annotated post-translational modification sites may not be a major determinant of *YWHAG* variant pathogenicity in our cohort (Table 1).

**FIGURE 1:**
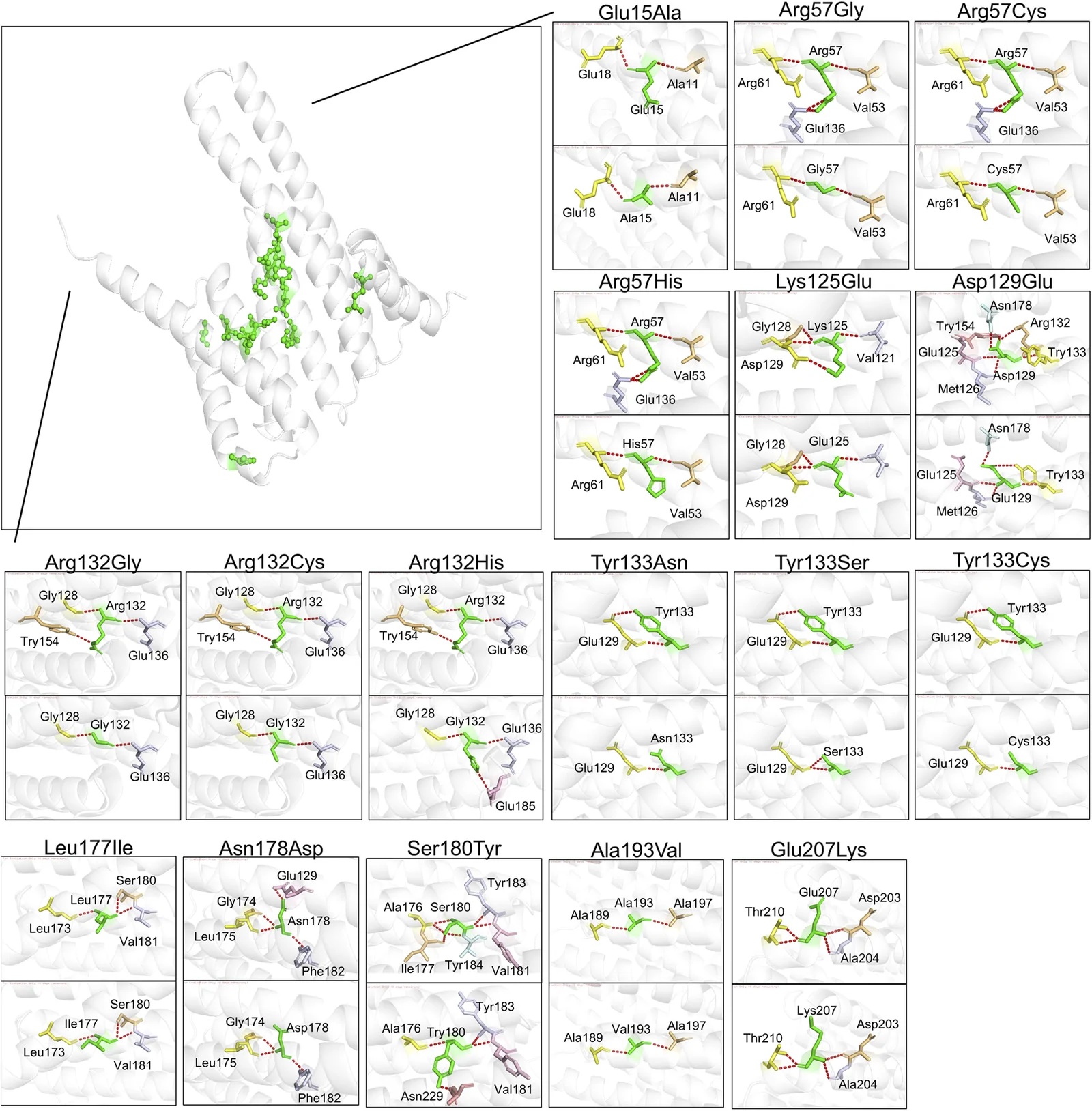
Hydrogen bond changes of *YWHAG* variants. The Hydrogen bond changes of disease-associated *YWHAG* variants.

**FIGURE 2:**
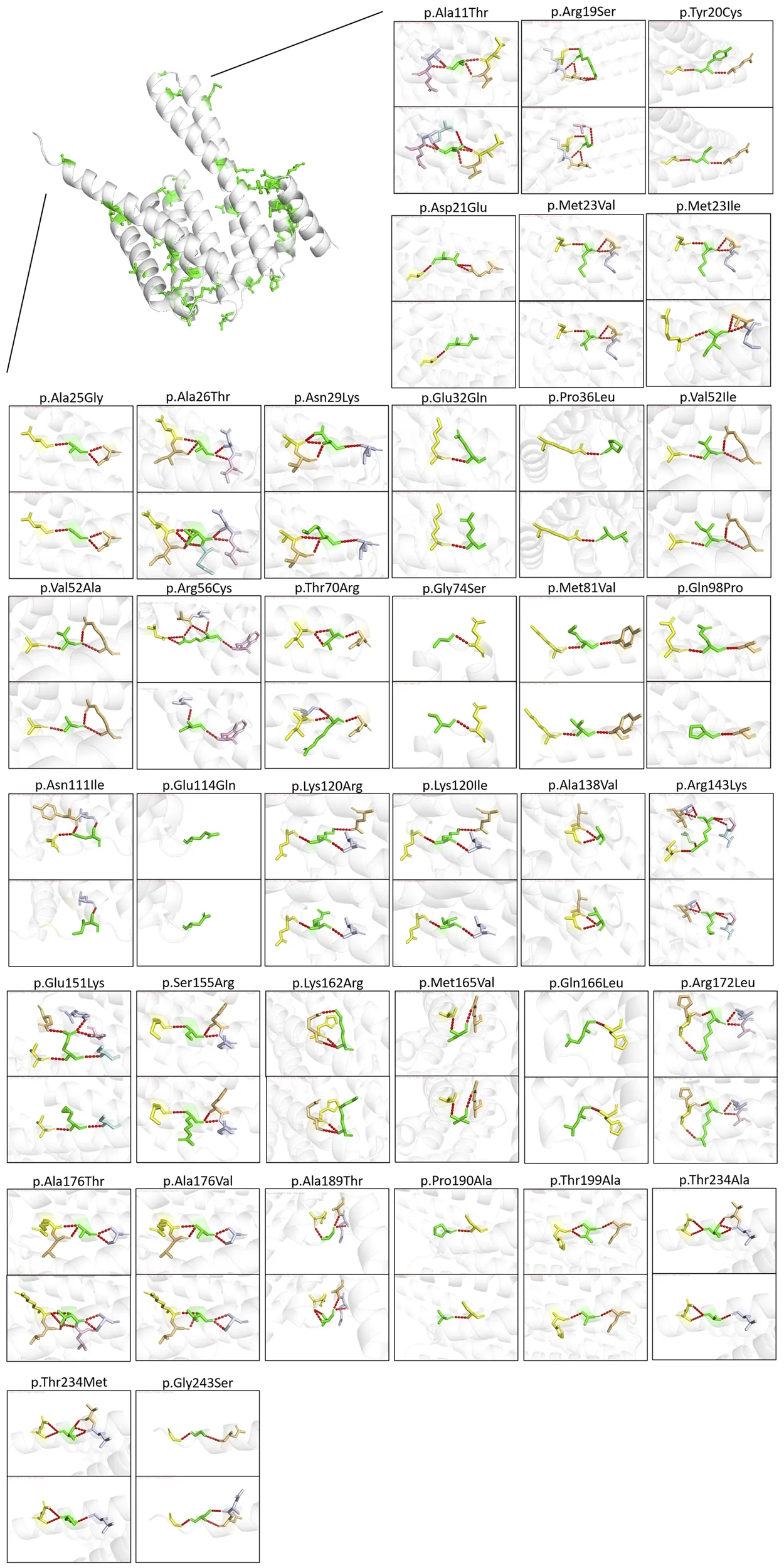
Hydrogen bond changes of *YWHAG* variants. The Hydrogen bond changes of control *YWHAG* variants. The red line represents hydrogen bonds.

**FIGURE 3:**
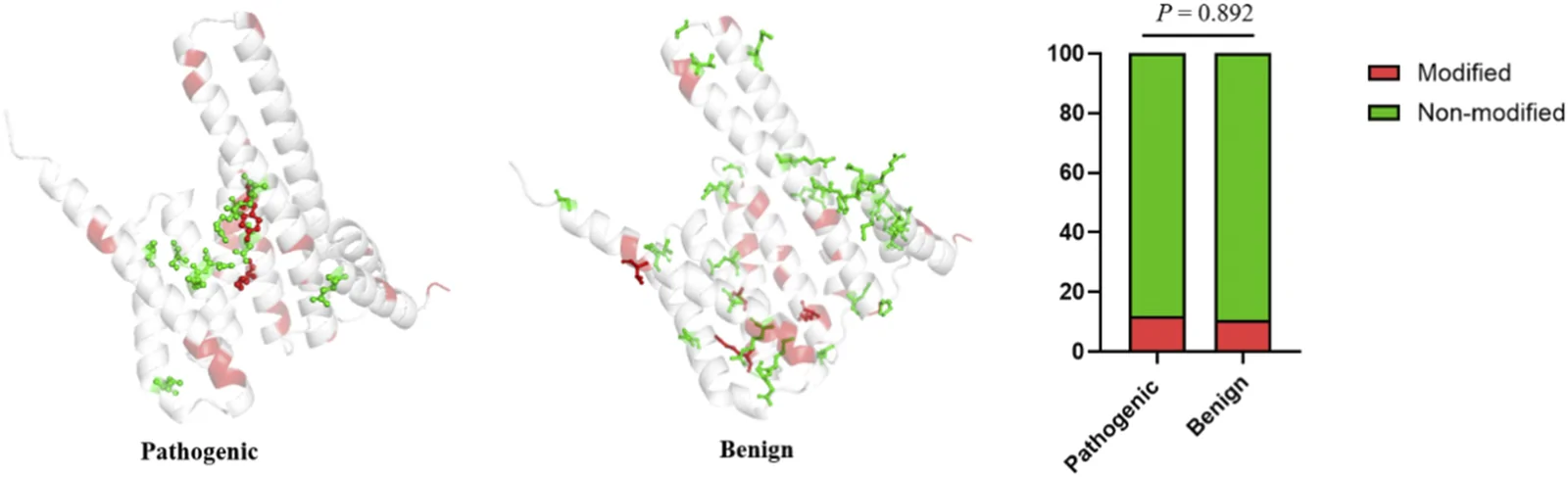
Structural localization of *YWHAG* variants and modified residue analysis. Left/middle: AlphaFold models of pathogenic and benign variants, with variant sites (green) and modified residues (red). Right: No significant difference in modified residue proportion between groups (P = 0.892).

**TABLE 1:** Modified residues of *YWHAG* variants.

No.	Modified residue
1	1
2	2
3	10
4	28
5	38
6	46
7	49
8	50
9	69
10	71
11	77
12	110
13	117
14	119
15	120
16	125
17	127
18	133
19	142
20	145
21	149
22	150
23	152
24	155
25	162
26	215
27	216
28	217
29	219
30	220
31	234
32	235

### 3.2 Spatio-temporal expression of *YWHAG*

The spatiotemporal pattern of gene expression serves as a fundamental underpinning for gene function. The *YWHAG* gene exhibits widespread expression across various tissues, with comparatively elevated levels detected in numerous brain regions (Figure 4A). Notably, YWHAG displays the highest expression levels in the developing brain, which is consistent with a potential role in neurodevelopmental processes (Figure 4B).

**FIGURE 4:**
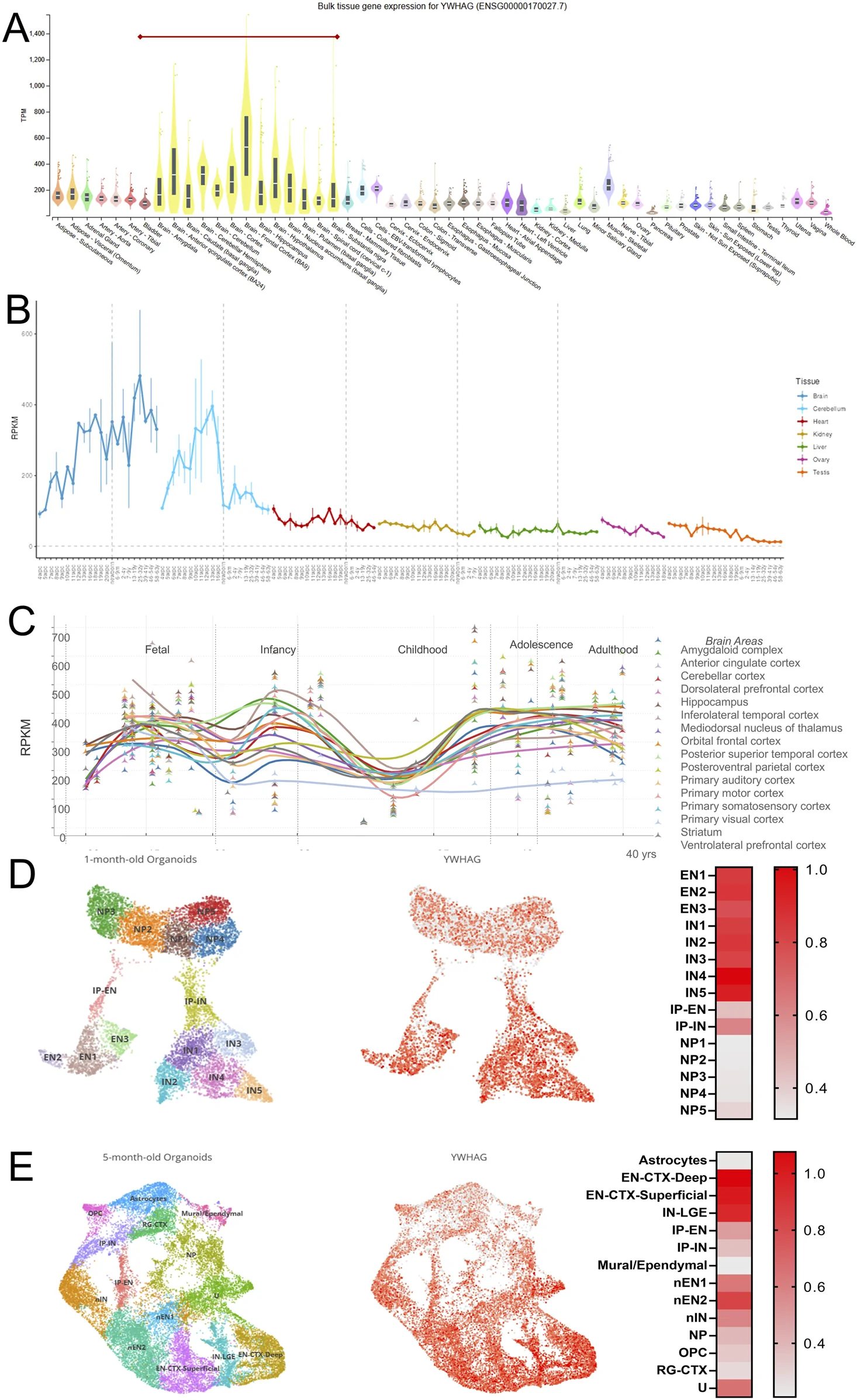
**(A)** RNA expression of *YWHAG* in normal adult tissues, sourced from the GTEx dataset. **(B)** Expression profile of *YWHAG* across organs at different developmental stages, sourced from the Evo-devo database. Abbreviations: wpc, weeks; postconception; m, months; y, years. **(C)** Expression profile of *YWHAG* across brain regions at different developmental stages. Abbreviations: pcw, post-conception weeks; mos, months; y, years. Data were obtained from the BrainSpan database. **(D,E)** The expression of *YWHAG* in the cells of single neural rosette-derived cortical organoids at 1 month and 5 months, sourced from the Human Organoid Single-Cell Browser. Abbreviations: EN, excitatory neuron; IN, inhibitory neuron; IP, intermediate progenitor; CTX, cortex; LGE, lateral ganglionic eminence; OPC, oligodendrocyte progenitor cell; RG, radial glial.

Further analysis of the temporal expression in various brain regions indicated that *YWHAG* is expressed throughout the lifespan, manifesting three peaks in early fetal, infancy, and adolescence (Figure 4C). YWHAG expression peaks during the early fetal stage, which may suggest a possible role in early neurodevelopment and a potential association with early seizure onset. Meanwhile, high YWHAG expression during adolescence appears to coincide with the period of relatively poor seizure control observed in some patients. Single-cell transcriptomic profiling of developing brain organoids demonstrated that *YWHAG* exhibited high expression levels in both inhibitory and excitatory neuronal populations at the 1-month stage. By contrast, at the 5-month stage, *YWHAG* expression became predominantly restricted to excitatory neurons (Figures 4D,E).

### 3.3 Protein‒protein interaction (PPI) network and enrichment analysis

To further investigate the molecular mechanisms and potential interactors of YWHAG, we constructed a Protein-Protein Interaction (PPI) network. As illustrated in Figure 5A, YWHAG is located within a highly interconnected core network. It demonstrates strong interactions with other members of the 14-3-3 protein family, including *YWHAB*, *YWHAE, YWHAQ*, and *YWHAZ*. Additionally, the network highlights critical interactions between YWHAG and key signaling kinases and cell cycle regulators, notably the RAF family (*ARAF*, *BRAF*, *RAF1*), CDC25 family phosphatases (*CDC25B*, *CDC25C*), YAP1, and BAD. This complex interaction web suggests that *YWHAG* may play an important role in mediating signal transduction and cellular regulation. To elucidate the biological significance of YWHAG and its interacting partners, we subsequently performed comprehensive enrichment analyses. Tissue expression (TISSUES) enrichment indicated that this gene set is prominently expressed in various malignancy-related cell lines, particularly cervical carcinoma and lymphocytic leukemia cells (Figure 5B). Gene Ontology (GO) analysis revealed the primary biological functions of the network. In terms of biological processes (BP), the network is significantly enriched in the regulation of phosphate metabolic processes, regulation of phosphorylation, the MAPK cascade, and the positive regulation of the G2/M transition of the meiotic cell cycle (Figure 5C). Consistently, molecular function (MF) analysis demonstrated strong enrichment in MAP kinase activity, enzyme binding, and phosphoserine residue binding (Figure 5D), which aligns well with the known scaffolding and phosphorylation-dependent binding functions of 14-3-3 proteins.

**FIGURE 5:**
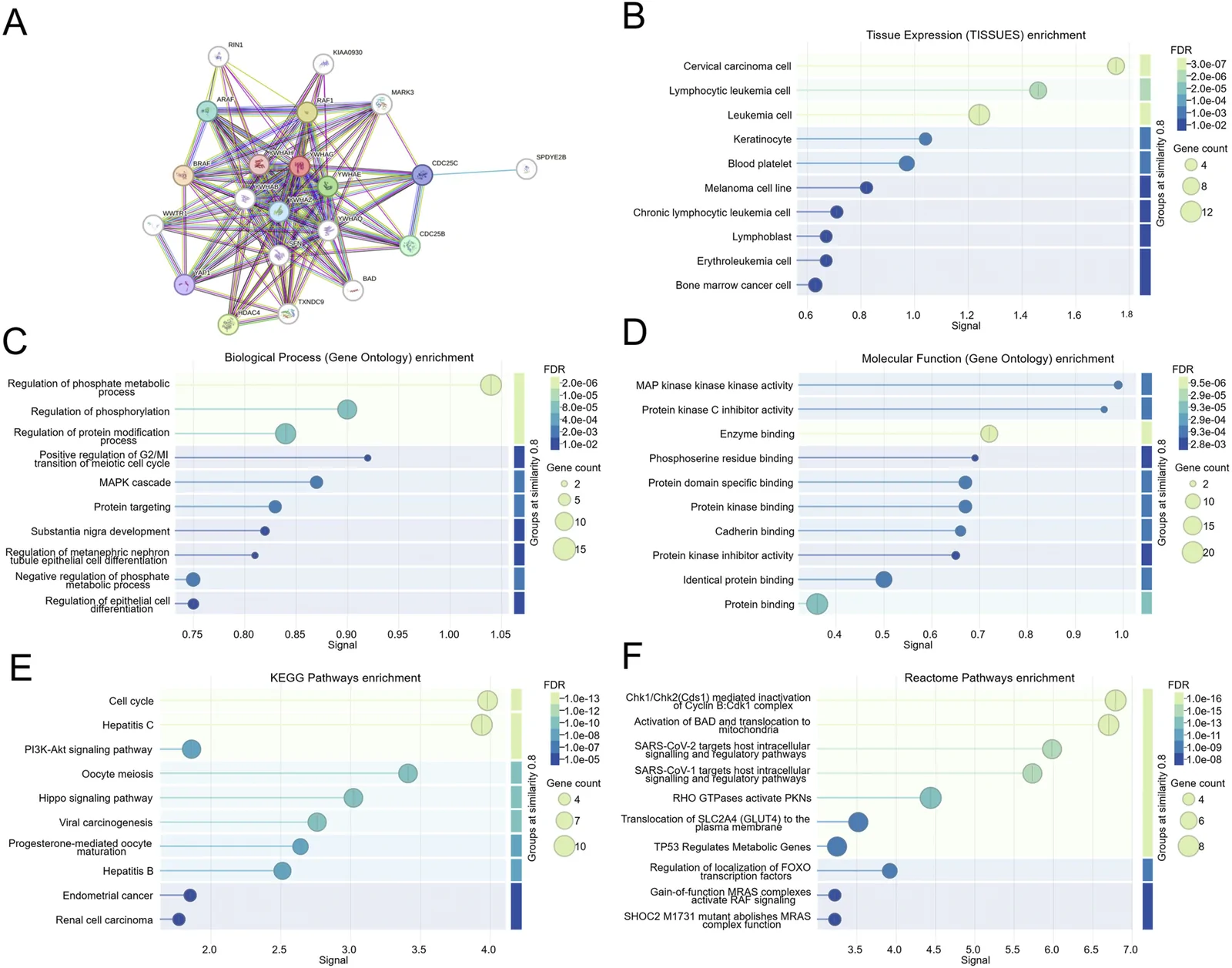
Functional annotations and phenotypic associations of *YWHAG*. **(A)** PPI network of YWHAG, showing core interactions with 14-3-3 family members, signaling kinases, and cell cycle regulators. **(B)** Tissue expression enrichment in malignancy-related cell lines. **(C,D)** GO enrichment for biological processes (phosphate metabolism, MAPK cascade, cell cycle regulation) and molecular functions (kinase activity, phosphoserine binding). **(E,F)** KEGG and Reactome pathway enrichment, highlighting roles in cell cycle, PI3K-Akt/Hippo signaling, and apoptosis.

Furthermore, KEGG pathway analysis showed that *YWHAG* and its interactors are primarily involved in the cell cycle, oocyte meiosis, the PI3K-Akt signaling pathway, and the Hippo signaling pathway (Figure 5E). Reactome pathway enrichment further refined these findings, specifically highlighting the network’s crucial involvement in cell cycle checkpoints, such as the Chk1/Chk2(Cds1)-mediated inactivation of the Cyclin B: Cdk1 complex, as well as apoptosis-related pathways like the activation of BAD and its translocation to mitochondria (Figure 5F). Taken together, these results showed the important role of *YWHAG* in regulating critical intracellular signaling cascades, kinase activity, and cell cycle progression.

### 3.4 Performance metrics of prediction algorithms

In this study, we systematically evaluated the performance of 34 bioinformatics pathogenicity prediction tools based on a rigorously curated dataset of 55 *YWHAG* gene missense variants, including 17 pathogenic variants and 38 benign control variants. Complete quantitative results are presented in Supplementary Table S4 and visualized in Figure 6. Core metrics, including Accuracy, Balanced Accuracy, Sensitivity, Specificity, Positive Predictive Value, Negative Predictive Value, Matthew’s Correlation Coefficient, and F1-score were comprehensively calculated. The results revealed significant differences in predictive performance among the tools, with MetaSVM achieving the highest Balanced Accuracy of 0.9280 and the highest Accuracy of 0.9455. Among them, MetaSVM exhibited the best overall performance with a Matthew’s Correlation Coefficient of 0.8710 and an F1-score of 0.9091, followed by MetaLR, REVEL, and MutFormer with excellent performance. Ensemble predictors generally outperformed individual predictors significantly. Twenty-two tools achieved 100% Sensitivity, and only MetaLR reached 100% Specificity. To further evaluate the robustness of these performance estimates and mitigate potential over-optimistic bias from gene-specific thresholds derived on the same dataset, we performed 1000-iteration bootstrap resampling with replacement for all 34 predictors. The bootstrap mean, standard deviation, and 95% confidence interval for each performance metric are provided in Supplementary Table S5.

**FIGURE 6:**
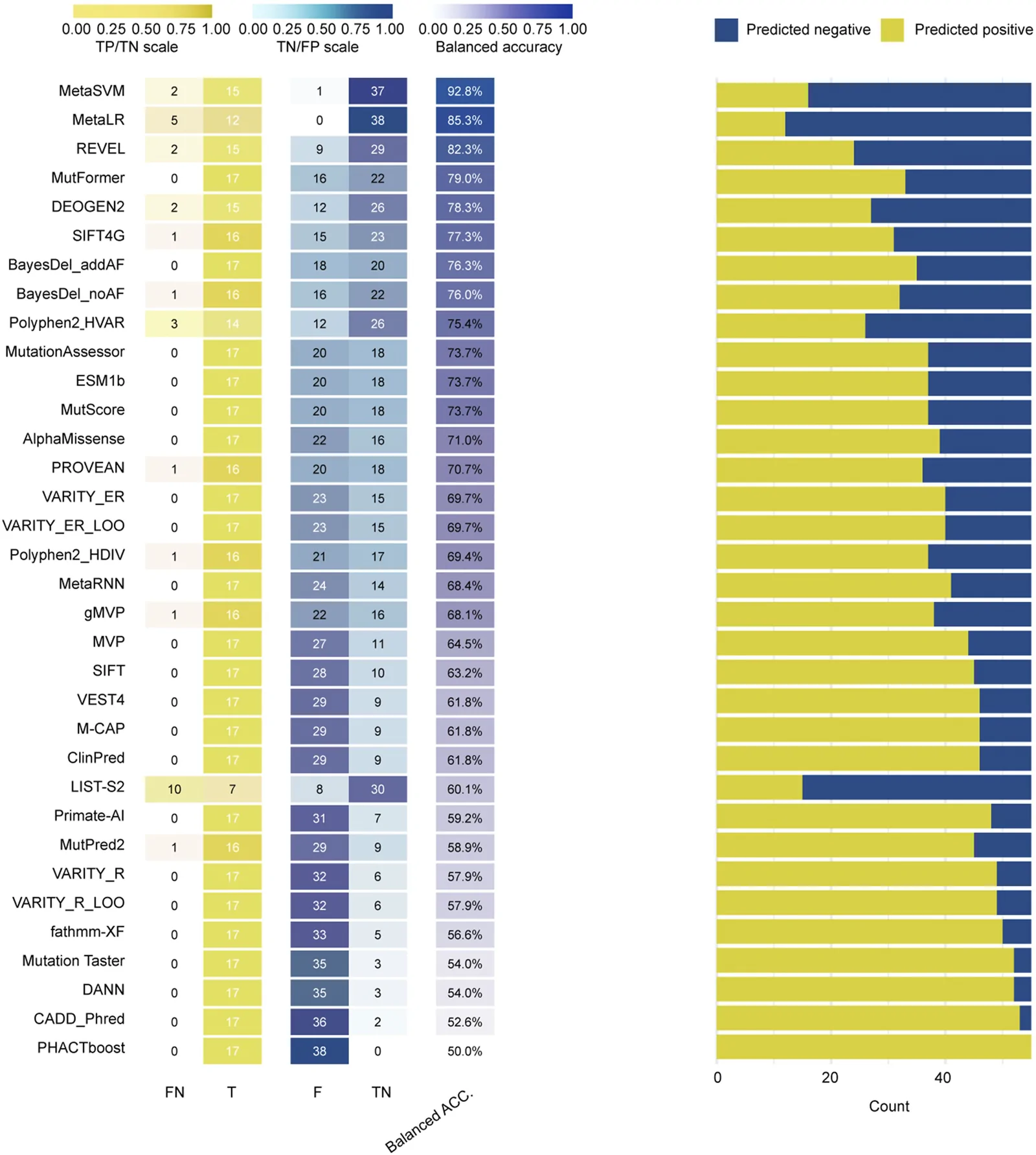
Performance evaluation of algorithms. For each tool, the left heat-map panel reports the true positives (TP) and false negatives (FN) for pathogenic variants and true negatives (TN) and false positives (FP) for control variants. The middle column gives the resulting balanced accuracy (balanced acc.), which was used to rank the tools from top to bottom. The right stacked bar shows the proportion of variants each algorithm used with recommended thresholds.

### 3.5 ROC curve analysis and AUC values

To visually present and compare the predictive performance of each variant pathogenicity prediction tool, receiver operating characteristic (ROC) curves were plotted in this study. The area under the curve (AUC), as a quantitative index of the ROC curve, can comprehensively reflect the diagnostic accuracy of a tool (Figures 7 and 8; Supplementary Table S6). Consistent with the above results, ROC curve analysis showed that ESM1b (AUC = 0.9861) performed the best, followed by MutationAssessor (0.9737), MetaSVM (0.9628), MetaLR (0.9582), ClinPred (0.9474), AlphaMissense (0.9466), MetaRNN (0.9396), BayesDel_addAF (0.9241), BayesDel_noAF (0.9241), and DEOGEN2 (0.9149), while LIST-S2 (AUC = 0.6416) had the lowest AUC value. These AUC values indicate that deep-learning and ensemble tools such as ESM1b, MutationAssessor, MetaSVM, MetaLR, ClinPred, AlphaMissense, and MetaRNN exhibit excellent discriminative ability for pathogenic variants and control variants in the *YWHAG* gene dataset.

**FIGURE 7:**
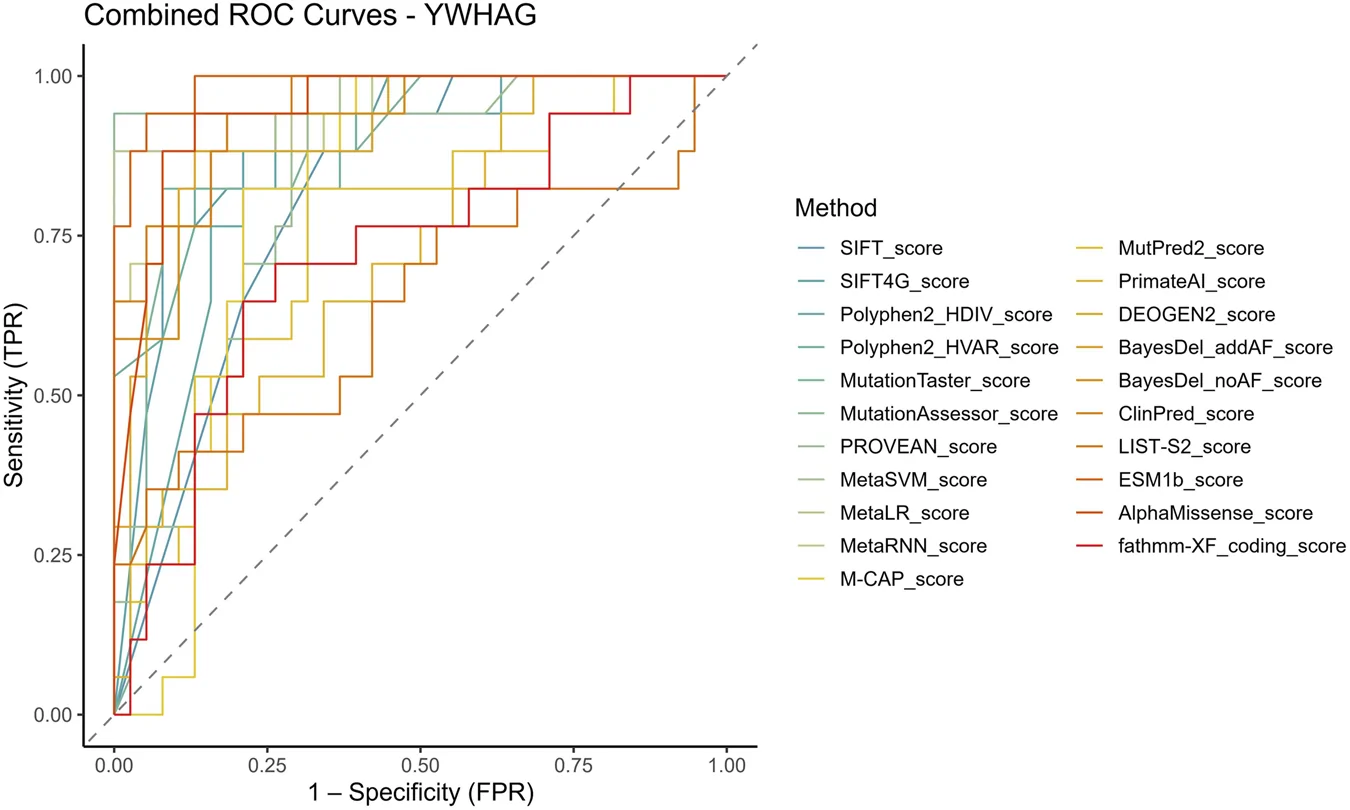
Receiver operating characteristic curve (ROC) performance and optimal thresholds of *in silico* tools for *YWHAG* variants. Combined ROC curves showed each color is one tool, and the dashed line meant no discrimination. All curves are provided in Supplementary Table S6.

**FIGURE 8:**
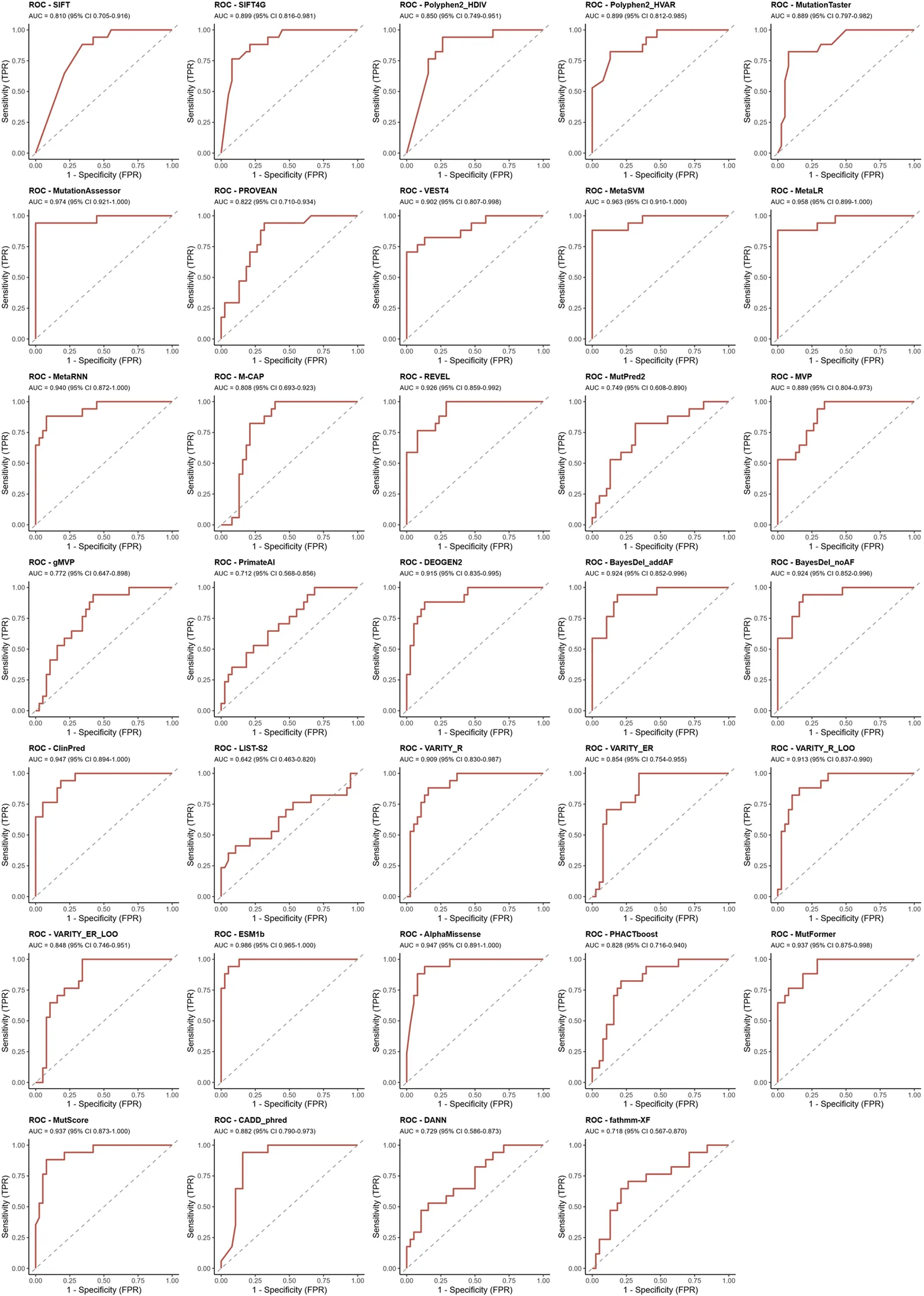
All ROC curves across thirty-four algorithms. ROC curves were presented with AUC and 90%CI, including SIFT, SIFT4G, Polyphen2_HDIV, Polyphen2_HVAR, MutationTaster, MutationAssessor, PROVEAN, VEST4, MetaSVM, MetaLR, MetaRNN, M-CAP, REVEL, MutPred2, MVP, gMVP, PrimateAI, DEOGEN2, BayesDel_addAF, BayesDel_noAF, ClinPred, LIST-S2, VARITY_R, VARITY_ER, VARITY_R_LOO, VARITY_ER_LOO, ESM1b, AlphaMissense, PHACTboost, MutFormer, MutScore, CADD_phred, DANN and fathmm-XF.

### 3.6 Between-group comparison of score distributions

In this study, we predicted continuous pathogenicity scores from 34 *in silico* prediction tools, classified all 55 *YWHAG* missense variants into disease-associated and benign control groups, and visualized the detailed score distribution of each individual tool between the two groups using integrated violin, box and scatter plots (Figure 9). The results demonstrated that 33 out of 34 tools showed statistically significant between-group score differences, with 24 tools reaching the strict threshold of high statistical significance (P < 0.0001). LIST-S2 failed to show a statistically significant difference (P = 0.0974), while fathmm-XF_coding (P = 0.0105) and PrimateAI (P = 0.0129) only exhibited weak to moderate statistical significance. Twenty-three tools displayed distinct separation patterns with nearly no detectable score overlap between groups, demonstrating excellent discriminative ability between *YWHAG* pathogenic and benign variants. In contrast, LIST-S2, fathmm-XF_coding, PrimateAI, and five other tools presented markedly broader and heavily overlapping distributions, indicating limited discriminative capacity and highlighting substantial performance disparities across distinct prediction algorithms for *YWHAG* missense variant pathogenicity assessment.

**FIGURE 9:**
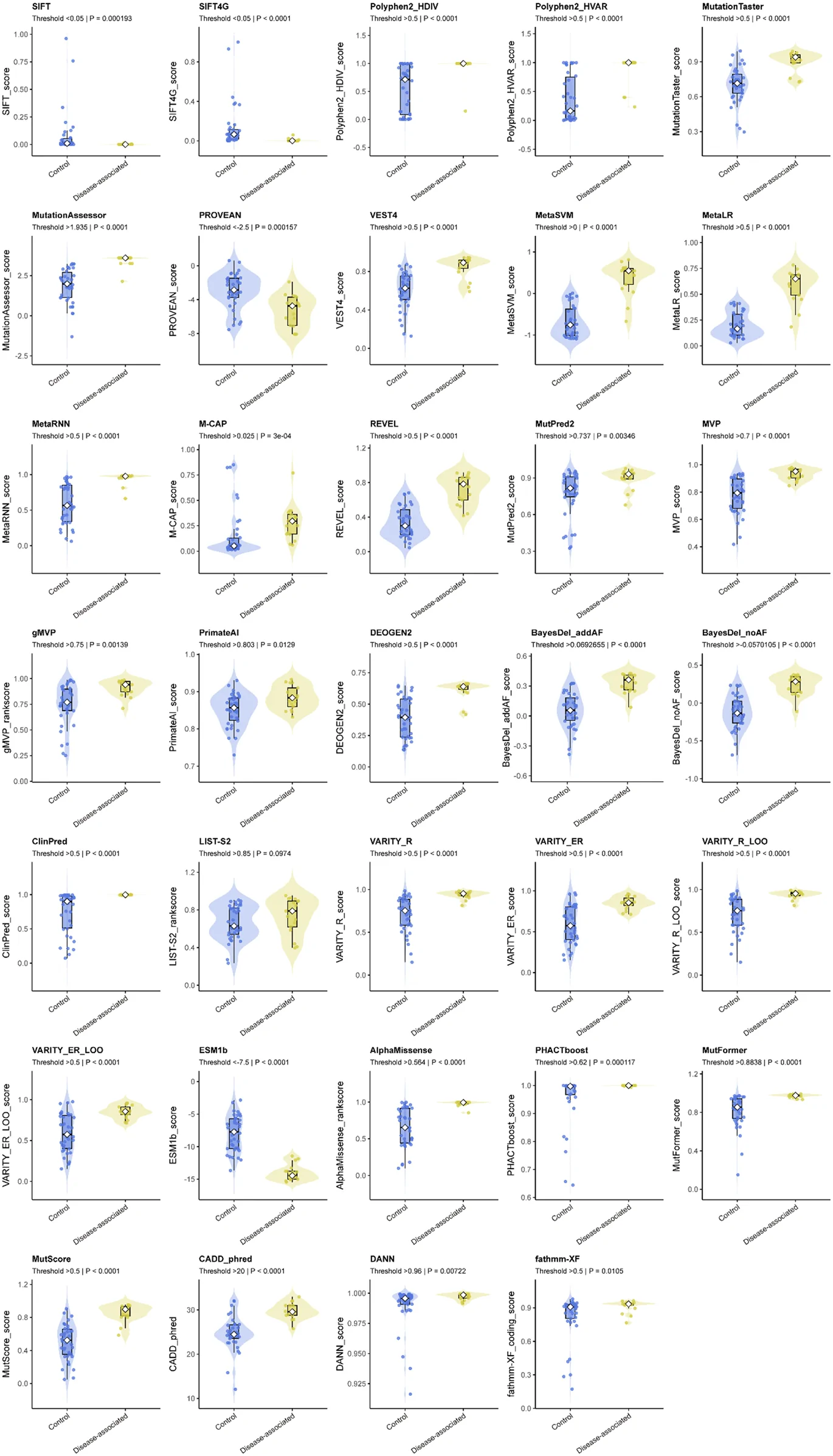
The distribution of *in silico* prediction raw scores for *YWHAG* variants. For each algorithm (including SIFT, SIFT4G, Polyphen2_HDIV, Polyphen2_HVAR, MutationTaster, MutationAssessor, PROVEAN, VEST4, MetaSVM, MetaLR, MetaRNN, M-CAP, REVEL, MutPred2, PrimateAI, DEOGEN2, BayesDel_addAF, BayesDel_noAF, ClinPred, LIST-S2, ESM1b, AlphaMissense, and fathmm-XF_coding), raw scores are shown for control (blue) and pathogenic (yellow) variants. Violin plots depict the score distribution. Higher scores indicate more deleterious predictions. Group differences were tested using a two-sided Wilcoxon rank-sum test; the classification threshold and corresponding P value are labeled above each panel.

## 4 Discussion

The *YWHAG* gene encodes 14-3-3γ, a highly conserved scaffold protein that is highly expressed in the central nervous system. Through phosphorylation-dependent protein–protein interactions, YWHAG regulates multiple downstream key signaling pathways (including MAPK cascades, protein phosphorylation regulation, apoptosis, and cell cycle checkpoints), thereby playing a central role in neuronal migration, synaptic plasticity, ion channel function, and the maintenance of excitation-inhibition balance (Amato et al., 2024; Vilmont et al., 2025). In this study, we systematically collected 55 *YWHAG* missense variants: 17 disease-associated variants classified as pathogenic/likely pathogenic by ACMG criteria, and 38 benign control variants retrieved from gnomAD with ClinVar-annotated benign/likely benign classifications and a minor allele frequency below 0.01. Combining protein structural modeling, spatiotemporal expression analysis, single-cell transcriptomics, PPI network construction, and comprehensive performance evaluation of 34 *in silico* pathogenicity prediction tools, we may help to further elucidate the potential molecular basis of *YWHAG* missense variants in the pathogenesis of DEE, and provide preliminary gene-specific *in silico* evidence for the clinical interpretation of genetic variants. In this study, we performed protein structure modeling using I-TASSER and structural visualization with PyMOL, and found that 64.7% of pathogenic missense variants caused alterations in the protein hydrogen bond network, while 52.6% of benign control variants exhibited similar hydrogen bond perturbations, with no statistically significant difference between the two groups. This finding demonstrates that qualitative structural analysis based solely on hydrogen bond changes cannot effectively distinguish pathogenic from benign missense variants in *YWHAG*. As a highly conserved scaffold protein, it exerts its functions through multiple structural aspects, including the conformation of the ligand-binding groove, the stability of the dimer interface, hydrophobic interactions, and electrostatic potential distribution, rather than relying exclusively on the hydrogen bond network (Yang et al., 2006; Obsilova and Obsil, 2022). Therefore, conventional three-dimensional structural modeling alone is insufficient to meet the demand for accurate interpretation in clinical genetic diagnosis, and there is an urgent need to integrate quantitative bioinformatics functional prediction tools for comprehensive evaluation, which constitutes the core motivation for conducting the large-scale benchmarking of tool performance in the present study. This limitation arises because predictive models cannot accurately define the functional domain boundaries of the YWHAG protein.Our modified residue analysis confirms that disruption of these annotated sites does not explain *YWHAG* variant pathogenicity in our cohort. These findings collectively suggest that conventional structural analyses alone are insufficient for reliable pathogenicity stratification, highlighting the utility of comprehensive *in silico* tools.

The spatiotemporal expression pattern of a gene can provide a useful basis for understanding its physiological function and disease phenotype. In this study, we observed that *YWHAG* appears to be broadly expressed throughout the body but shows relatively high expression in multiple brain regions, with three apparent expression peaks across the human lifespan: the early fetal stage, infancy, and adolescence. The expression peak in the early fetal stage is broadly consistent with the seizure onset as early as 1 month of age reported in some patients with DEE, raising the possibility that *YWHAG* may be involved in early neurogenesis, neuronal migration, and neural circuit formation. The expression peak in adolescence may coincide with a period when seizure control appears relatively poor in some patients at this developmental stage. Single-cell transcriptomics further suggested that *YWHAG* is expressed at relatively high levels in both excitatory and inhibitory neurons in 1-month-old cerebral organoids, whereas its expression appears to be mainly restricted to excitatory neurons at 5 months of age, suggesting a possible role in maintaining the excitation-inhibition balance. These observations of relatively high brain expression and a dynamic spatiotemporal pattern are consistent with the possibility that *YWHAG* mutations may contribute to abnormalities in neuronal migration, synaptic plasticity, and ion channel regulation, which could potentially lead to refractory epilepsy and developmental delay. PPI network analysis revealed that 14-3-3γ encoded by *YWHAG* lies at the core of a highly interconnected signaling regulatory network; it not only forms homodimers/heterodimers with other YWHAG family members such as YWHAB and YWHAE, but also interacts strongly with key signaling molecules including the RAF kinase family, CDC25 phosphatase family, YAP1, and BAD. Functional enrichment analysis further indicated that these interacting molecules are mainly involved in the regulation of phosphate metabolism, MAPK cascade, cell cycle checkpoints, and the PI3K-Akt and Hippo signaling pathways. YWHAG binds to target proteins in a phosphorylation-dependent manner to regulate their subcellular localization, stability, and enzymatic activity, thereby affecting neuronal migration, synaptic plasticity, ion channel function, and cell survival. Missense variants in *YWHAG* may disrupt these critical protein interactions, leading to disorders in downstream signaling pathways and ultimately triggering neurodevelopmental abnormalities and epileptic seizures. These findings may help to clarify the molecular pathway basis of *YWHAG*-induced DEE and provide potential targets for subsequent targeted therapy research.

In this study, we conducted a comprehensive performance benchmarking of 34 clinically commonly used pathogenicity prediction tools for missense variants based on 55 rigorously quality-controlled *YWHAG* missense variants. The results revealed significant differences in the discriminative efficacy among these tools. Among the ensemble learning tools, MetaSVM showed the optimal overall performance, with an accuracy of 94.55%, a balanced accuracy of 92.80%, and the highest Matthews correlation coefficient (0.8710) and F1 score (0.9091); MetaLR achieved 100% specificity and positive predictive value with a balanced accuracy of 85.29%, demonstrating outstanding capability in discriminating benign variants. The deep learning tool ESM1b attained an extremely high AUC value of 0.9861, the highest among all tools, and AlphaMissense (AUC = 0.9466) as well as the ensemble tools REVEL and MutFormer also exhibited excellent performance in distinguishing pathogenic from benign variants. In contrast, tools including LIST-S2, CADD_phred, and DANN showed markedly poor performance: LIST-S2 presented no statistically significant difference in prediction scores between the two groups (P = 0.0974) with a balanced accuracy of only 60.06%, so it is not recommended for the pathogenicity assessment of YWHAG missense variants. Traditional sequence conservation-based tools (such as SIFT and Polyphen2) had high sensitivity but generally insufficient specificity, which were prone to false-positive results and had limitations in clinical single application. Furthermore, we calculated the gene-specific optimal thresholds of these high-performance tools for *YWHAG* using Youden’s index, which can accurately calibrate the strength of PP3/BP4 evidence in the ACMG guidelines for genetic interpretation and significantly improve the accuracy and reliability of clinical pathogenicity interpretation of *YWHAG* missense variants (Pejaver et al., 2022).

Moreover, given that meta-predictors and ensemble tools including MetaSVM, MetaLR and REVEL are commonly trained on large variant repositories such as ClinVar and HGMD, we comprehensively assessed the potential circular bias arising from training dataset overlap, with all chronological details summarized in Supplementary Table S3. The 34 prediction tools used in this study were developed between 2010 and 2025, with 73% released before 2021. Prior to the development of these core tools, consistent and authoritative criteria for assessing the pathogenicity of *YWHAG* variants were unavailable, so none of our *YWHAG* variants were included in the original training datasets or participated in core model construction. Only two benign variants (p.Lys120Arg reported in 2023 and p.Lys162Arg reported in 2024) were incorporated into the updated training sets of a small number of tools, and no pathogenic *YWHAG* variants had been deposited in public databases for training before their publication, confirming all functionally relevant variants in our cohort were identified after core tool development. Collectively, there is almost no significant overlap between our test dataset and the original training datasets, which helps mitigate potential interference from model data memorization. The favorable performance of these tools likely reflects their genuine biological generalization capacity rather than overfitting to known variants. This study has several limitations. First, the limited sample size reduces the statistical power for evaluating the performance of prediction tools. Second, our analyses are restricted to bioinformatic predictions without experimental validation in cellular or animal models. Third, although we performed 1000-iteration bootstrap resampling to assess the robustness of performance metrics and mitigate over-optimistic bias from thresholds derived on the same dataset, the gene-specific optimized cut-offs were derived exclusively from the present dataset and lack independent external validation. Accordingly, their utility for formal ACMG/AMP evidence strength assignment remains preliminary, and further confirmation in larger independent *YWHAG* variant cohorts is required.

## Data Availability

The original contributions presented in the study are included in the article/Supplementary Material, further inquiries can be directed to the corresponding authors.
